# Hemilaterally masked arterial spin labeling by intentional magnetic field changes in the labeling area due to placement of material with high susceptibility

**DOI:** 10.1371/journal.pone.0200648

**Published:** 2018-07-12

**Authors:** Hiroaki Hagiwara, Yoshito Nakajima, Tadashi Ikegami, Yoshinori Kinno, Megumi Kumada

**Affiliations:** Department of Radiology, Yokohama Minamikyosai Hospital, Yokohama,Kanagawa, Japan; Universitatsklinikum Freiburg, GERMANY

## Abstract

**Background and purpose:**

Arterial spin labeling(ASL)with magnetic resonance imaging (MRI) is an effective method for estimating cerebral blood flow (CBF). Furthermore, assessing perfusion territories of arteries is useful for determining the treatment strategy of patients with carotid artery stenosis. ASL with selective vessel labeling is an effective method to obtain perfusion mapping, however, the application for selective labeling is not installed on all MR scanners. The purpose of this study is to establish a method to selectively mask in the labeling area using material with high susceptibility instead of selectively labeling to obtain a partial perfusion image.

**Materials and methods:**

ASL perfusion images were performed in five volunteers. Masking was applied by placing a stainless-steel bolt and nuts on the neck. The area of artifacts extended to the carotid artery was confirmed by the localizer image. In the obtained masked ASL, blood flow of the left and right cerebrum and cerebellum was measured and compared with control ASL without masking. By subtracting masked ASL from the control ASL, the perfusion territory of the carotid artery on the masked side was identified.

**Results:**

Mean CBF which was 39.6 ml/(100 g × min) in control ASL decreased to 16.1 ml/(100 g × min) in masked ASL, and the masking ratio was 59.6%. There were no significant differences in the CBF of non-masked areas under the control ASL condition (39.6± 5.2 ml/[100 g × min]) btween that under the masked ASL condition (39.4 ± 7.0 ml/[100 g × min]). By subtracting masked ASL from control ASL, we successfully visualized the hemilateral carotid artery’s perfusion territory.

**Conclusion:**

Intentional susceptibility artifacts with non-magnetic metals on the neck can mask spin labeling of the carotid artery. Furthermore, hemilateral carotid artery perfusion territories can be visualized in hemilaterally masked ASL.

## Introduction

Arterial spin labeling (ASL) with magnetic resonance imaging (MRI) is an effective method for estimating cerebral blood flow (CBF). Unlike dynamic susceptibility contrast perfusion with MRI, which requires a rapid injection of a contrast agent tracer, ASL uses an intrinsic tracer by magnetically labeling of arterial blood. Depending on the labeling method, ASL is classified as pulsed, continuous, pseudocontinuous (pCASL), or velocity-selective.[1]

Assessing perfusion territories of brain arteries is useful for determining treatment strategies for patients with carotid artery stenosis. [2] The collateral circulation via the circle of Willis and leptomeningeal anastomosis plays a pivotal role in the pathophysiology of cerebral ischemia. Several techniques have been developed to visualize the perfusion territories by selective arterial labeling.[3, 4] However, the application for this technique is not installed on all MRI scanners.

When performing ASL, clinicians should be aware of several artifacts that may affect diagnostic accuracy. Susceptibility variations in the labeling plane can dephase arterial blood protons, thereby disrupting the conditions required for pseudocontinuous inversion, causing poor or absent labeling.[5] This phenomenon occurs frequently in patients with dentures and in patients with carotid artery stents. We noticed that selective ASL images can be taken if this phenomenon is caused intentionally. Thus we developed hemilaterally masked ASL, which involves pCASL with placement of material with high susceptibility in the labeling area, and evaluated this technique’s feasibility for territory mapping in healthy volunteers.

## Methods

Our study protocol was approved by the Ethics Committee of Yokohama Minamikyosai Hospital and adhered to the principles of the Declaration of Helsinki. All subjects provided written informed consent. We scanned five volunteers (three men and two women; mean age: 27.0 years; age range: 21–31 years) without known cerebrovascular diseases.

We conducted ASL perfusion imaging with a 3-tesla MRI device (Discovery 750w 3.0T; GE Healthcare, Chicago, IL, USA). We used a whole-brain three-dimensional spiral fast-spin echo sequence (six spiral arms of 1004 points each; z-direction phase encoding = 32; section thickness = 4 mm; TR = 5.357 s; post-label wait time = 1.525 s; NEX = 1; total acquisition time = 2 min, 29 s) with background suppression and a pseudocontinuous scheme.

**Fig 1:**
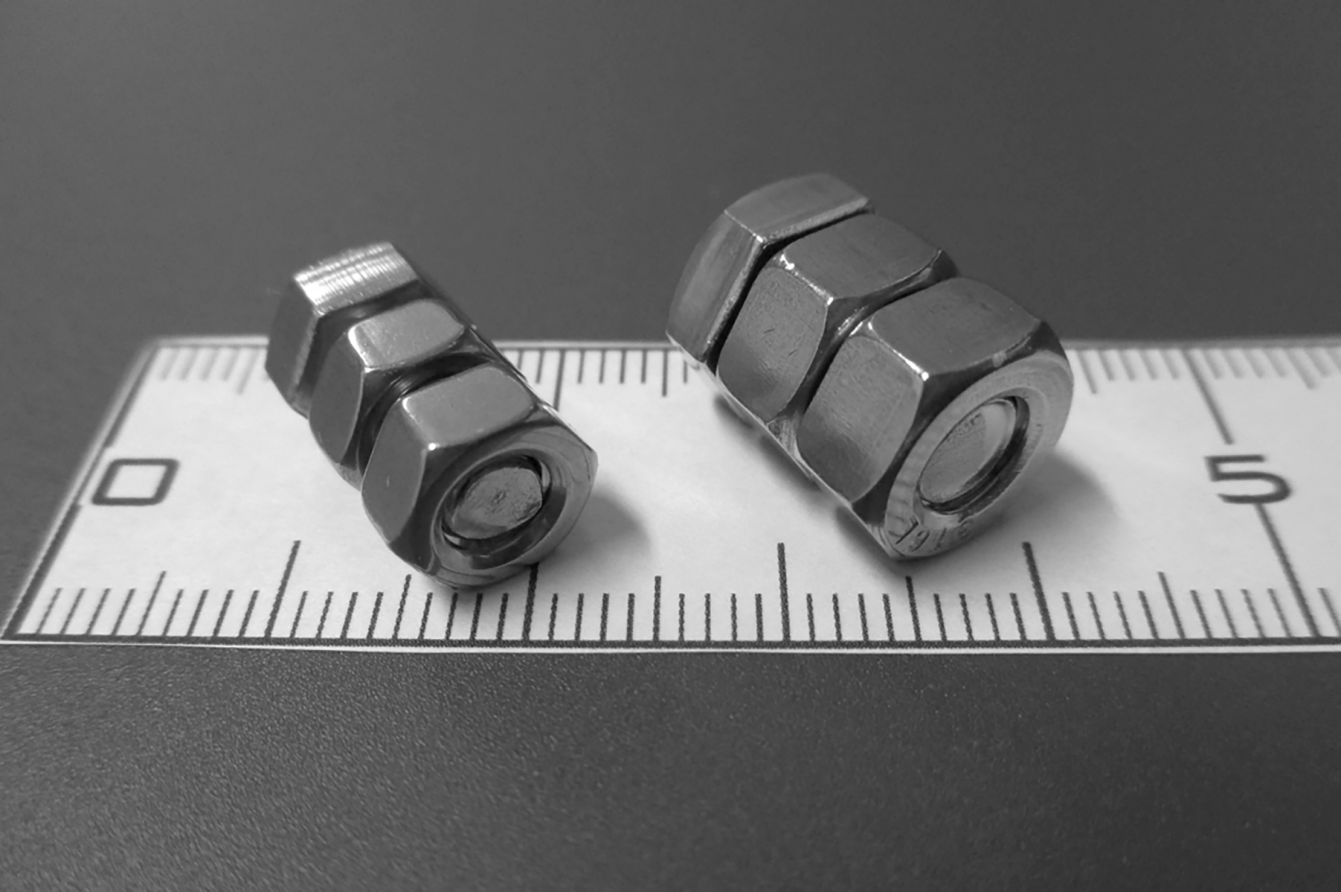
Bolt and nuts for masking made of ASTM standard grade 316L stainless steel.

To generate a susceptibility artifact and mask spin labeling, we used a hex bolt and two nuts made of grade 316L ASTM standard stainless steel. For most subjects, we used a large set in which the bolt had a 10-mm head diameter and a 14-mm shaft length (ISO M6), but for subjects who weighed less than 50 kg, we used a smaller set in which the bolt had an 8-mm head diameter and a 13-mm shaft length (ISO M5) (Fig 1). Smaller bolts were used in lighter subject as it was found in preliminary scans that the distance from the skin to the target carotid artery was smaller in these subjects and the small artifact area generated by the smaller bolt was sufficiently reached. The force applied to the stainless-steel bolt and nuts was measured by ASTM force measurement method (F2052-06) at edge of the MRI bore where the magnetic field was strongest.

**Fig 2:**
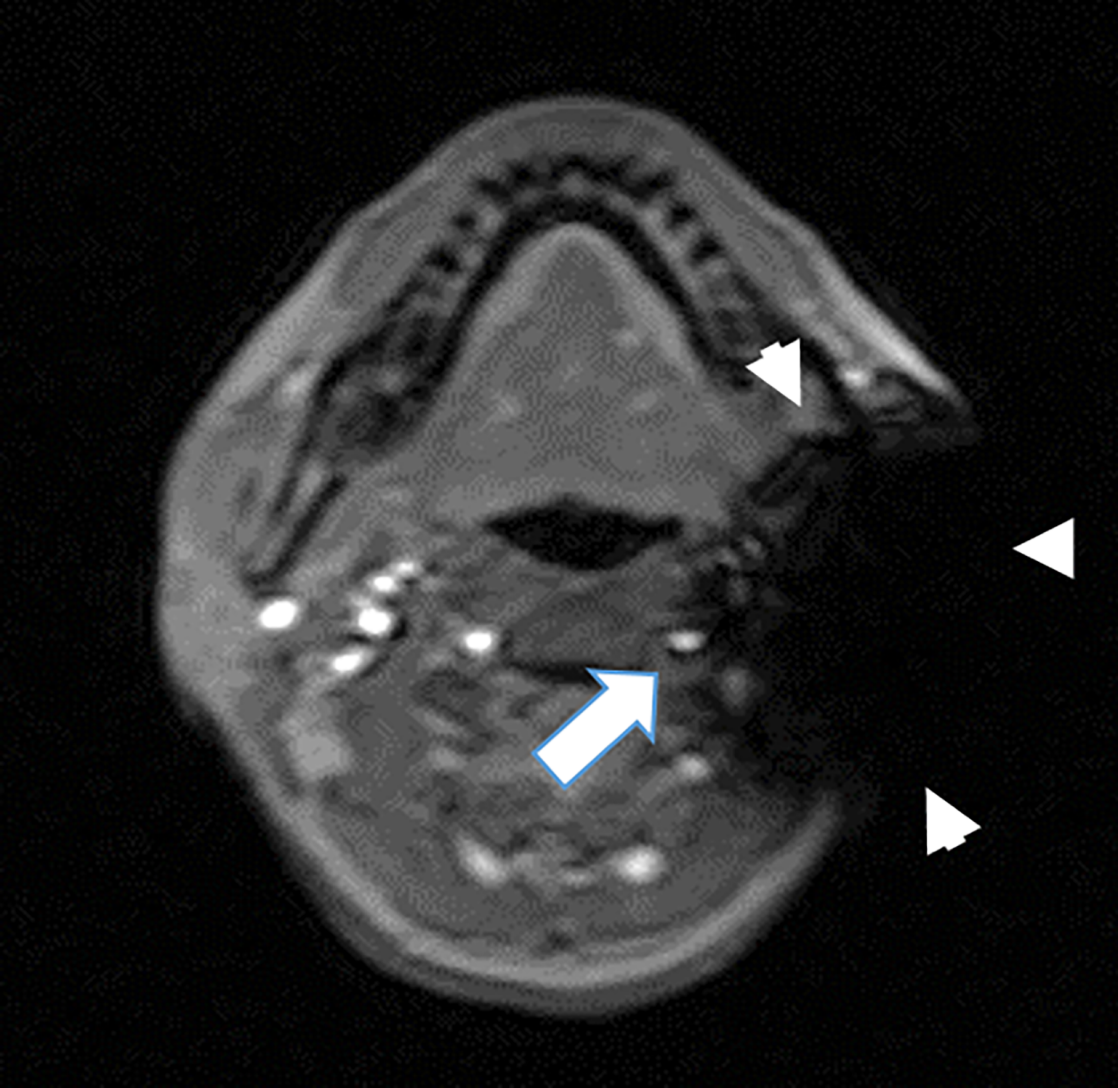
Localizer image showing the metal-induced susceptibility artifact extending to the ICA (arrowheads). The ipsilateral vertebral artery (arrow), contralateral ICA, and vertebral artery are unaffected.

Based on the localizer image, the labeling plane was set 4 cm caudal from the lower cerebellar border, and the tape-wrapped bolt and nuts were placed on the mandibular angle. In the localizer images (FGR; TR = 2.1 s, TE = 6.8 s, flip angle = 30), we confirmed that the bolt and nuts caused a susceptibility artifact area extending to the carotid artery but not to the vertebral artery (Fig 2).

We first obtained right-side masked ASL scans, followed by left-side masked ASL scans, and finally performed unmasked control ASL scans. We obtained subtraction ASL images for territory mapping by subtracting the masked ASL images from the control ASL images. We measured CBF and cerebellar blood flow (CeBF) levels within masked and unmasked areas. We calculated masking ratios with the following equation:

$$Masking\;ratio=(1-\frac{masked\;CBF}{Unmasked\;CBF})\times 100\%.$$

The data are reported as means ± standard deviations. We performed statistical comparisons with paired-samples t-tests in SPSS v. 21.0 for Windows (IBM, Armonk, NY, USA). We defined statistical significance as P < .05.

## Results

No adverse events occurred during the ASL scans. As measured by ASTM force measurement method, the shift angle of string caused by the magnetic force was 15 degrees for the large bolt and nuts and 14 degrees for the smaller one. Both shift angles were less than the standard 45 degrees that is considered safe, which is same as gravity, and thus, were also considered safe in this study.

**Fig 3:**
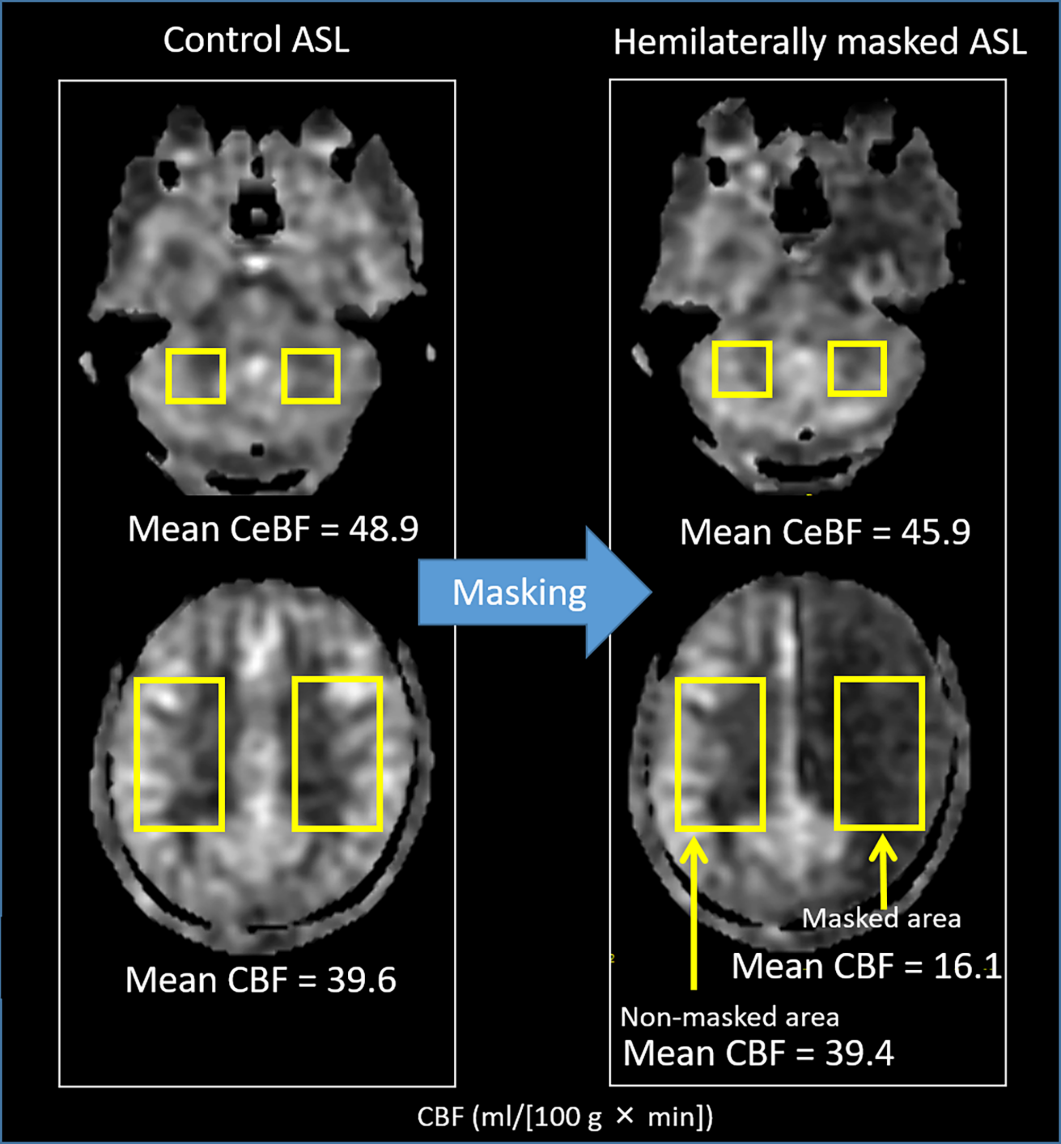
Rectangular ROIs for CBF and CeBF measurements covering the bilateral cerebrum and cerebellum in the control ASL and masked ASL images. Abbreviations: ASL, arterial spin labeling; CeBF, cerebellar blood flow.

In every hemilaterally masked ASL image, CBF was decreased in internal carotid artery (ICA) territories of the frontal, temporal, and parietal lobes on the masked side. Within rectangular ROIs covering both cerebellar hemispheres and the cerebral hemispheres above the upper end of the lateral ventricles, we found that the mean CBF and mean CeBF in control ASL scans were 39.6 ml/(100 g × min) and 48.9 ml/(100 g × min), respectively (Fig 3, Table 1). The mean CBF in the masked cerebral area was 16.1 ml/(100 g × min), and the mean masking ratio was 59.6%.

**Table 1:** CBF and CeBF (ml/[100 g × min]) under control ASL and masked ASL conditions. The numbers in parentheses are percentages relative to control ASL values.

	Control ASL				Right-masked ASL				Left-masked ASL			
	Cerebrum		Cerebellum		Cerebrum		Cerebellum		Cerebrum		Cerebellum	
Subject	Right	Left	Right	Left	Right	Left	Right	Left	Right	Left	Right	Left
1	43	38	57	64	16	41	58	59	44	14	63	53
					(37)	(108)	(102)	(92)	(102)	(37)	(111)	(83)
2	43	46	57	60	24	48	52	67	46	13	50	49
					(56)	(104)	(91)	(112)	(107)	(28)	(88)	(82)
3	35	28	40	32	9	27	30	31	28	10	37	31
					(26)	(96)	(75)	(97)	(80)	(36)	(93)	(97)
4	42	38	43	42	9	39	28	39	39	14	32	19
					(21)	(103)	(65)	(93)	(93)	(37)	(74)	(45)
5	43	40	48	46	24	43	47	54	39	28	51	58
					(56)	(108)	(98)	(117)	(91)	(70)	(106)	(126)

Abbreviations: ASL, arterial spin labeling; CeBF, cerebellar blood flow

In masked ASL scans, the mean CBF of the contralateral cerebral hemisphere’s non-masked area was 39.4 ml/(100 g × min). We detected no significant differences (t_(9)_ = 0.183, P = .86) between non-masked area CBF under the control ASL condition (39.6 ± 5.2 ml/[100 g × min]) and that under the masked ASL condition (39.4 ± 7.0 ml/[100 g × min]). We also detected no significant differences (t_(19)_ = 1.82, P = .08) between CeBF under the control ASL condition (48.9 ± 9.9 ml/[100 g × min]) and that under the masked ASL condition (45.4 ± 13.5 ml/[100 g × min]). These results suggest that the hemilaterally masked ASL technique effectively masks target-side labeling and does not affect contralateral CBF or CeBF.

**Fig 4:**
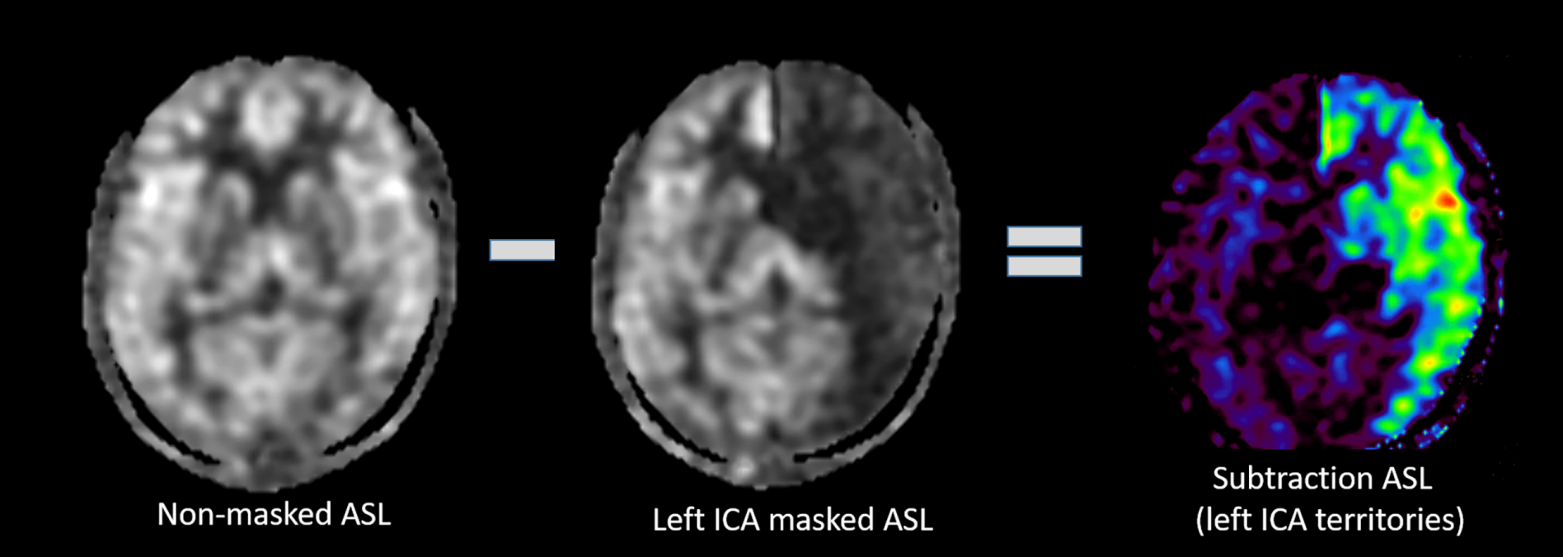
In the subtraction ASL image obtained by subtracting the masked ASL image from the control ASL image, masked-side ICA territories are clearly visualized. Abbreviations: ASL, arterial spin labeling.

**Fig 5:**
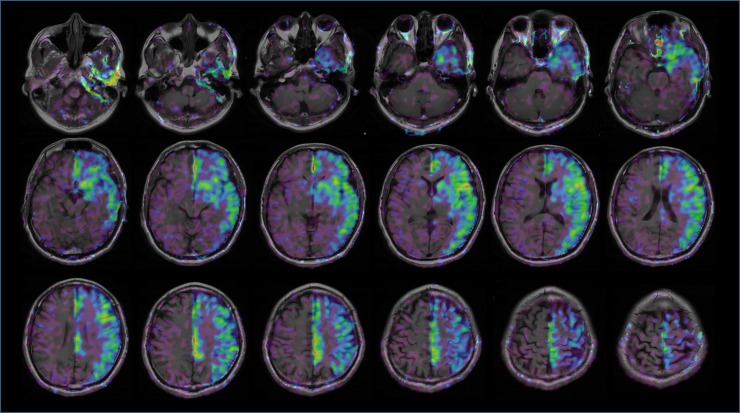
In fused T1WI and subtraction ASL images, the visualized left ICA perfusion area matches the known anatomical distribution. Abbreviations: ASL, arterial spin labeling.

In the subtraction ASL images, the ICA’s flow territories were clearly depicted, and almost no substantial contralateral CBF or bilateral CeBF was observed (Figs 4 and 5).

**Fig 6:**
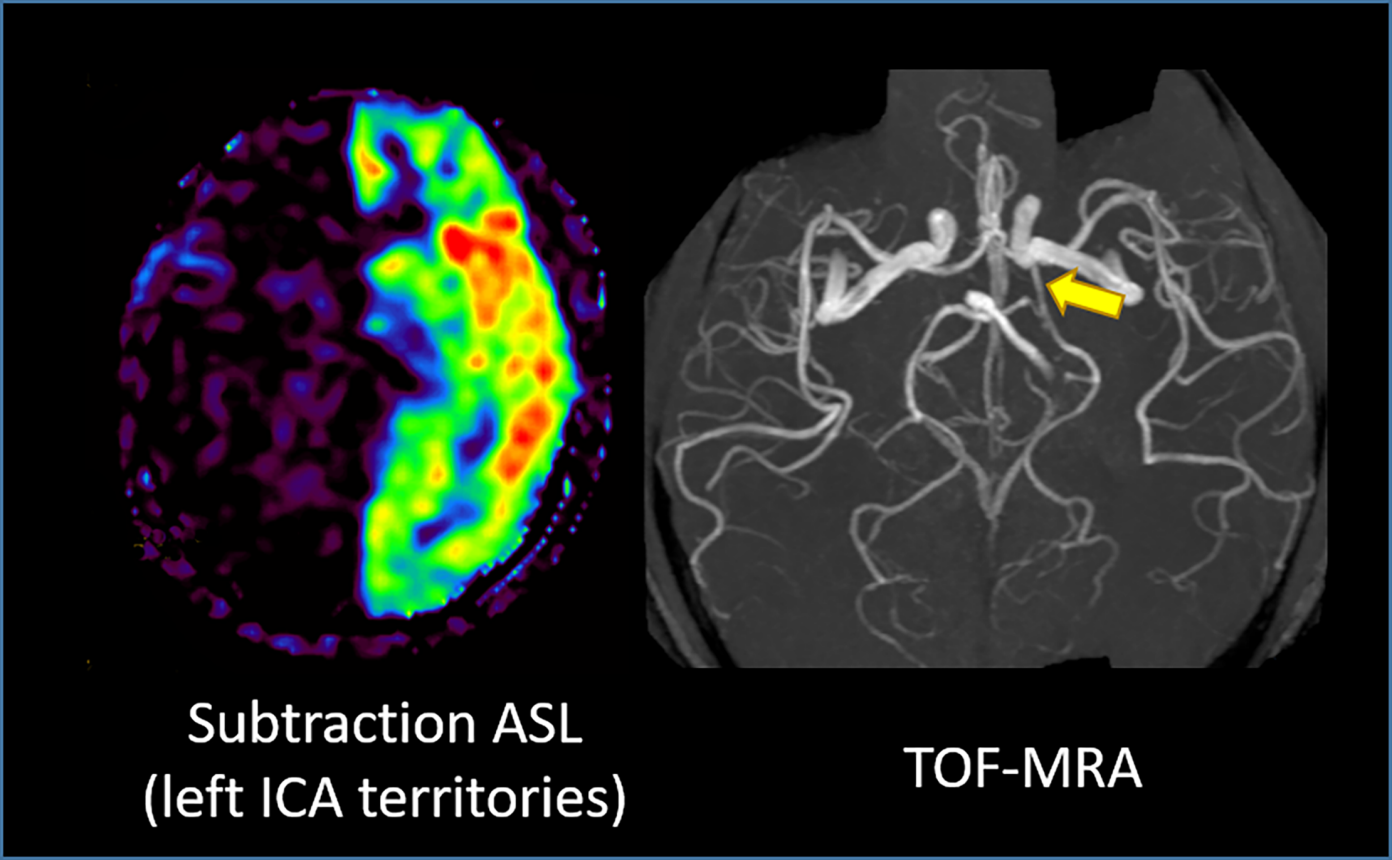
The left ICA perfusion area covers the entire left cerebrum in the subtraction ASL image. The TOF-MRA image reveals a well-developed left posterior communicating artery (arrow). Abbreviations: ASL, arterial spin labeling.

In one subject, the ICA perfusion area covered the entire left cerebrum (Fig 6). The TOF-MRA image revealed a well-developed left posterior communicating artery that perfused the posterior cerebral artery.

## Discussion

The present study aimed to determine whether it was possible to acquire flow territory maps of ICA by intentional susceptibility artifact. Susceptibility artifacts are troublesome phenomena that degrade MRI image quality and minimizing them is a serious clinical issue.[6] However, artifacts can be advantageous in certain diagnostic imaging methods. For example, chemical shift artifacts can aid in the detection of trace amounts of fat in adrenal adenomas.[7] Our results indicate that our imaging technique is another example in which artifacts are beneficial.

To efficiently mask spin labeling, appropriate metals must be suitably placed. Among the MRI-safe metals typically used in implanted medical devices, titanium alloys produce no artifacts, and cobalt-chromium alloys have too narrow a range and cannot mask the ICA even when placed on the neck surface.[8] However, stainless steel has an adequate artifact area and can achieve desired masking effects when appropriately positioned. In our experiments, the masking effect was weak if the artifact area did not extend to the carotid artery, whereas the CeBF and CBF of the posterior cerebral artery’s perfusion area were decreased if the artifact area reached the vertebral artery. Because the purpose of hemilaterally masked ASL is to depict the ICA’s perfusion area in subtraction ASL images, excessive masking causes disadvantageous masking of vertebral arteries. To obtain accurate hemilaterally masked ASL images, it is important to confirm an appropriate masking area with the localizer images.

It is often difficult to identify responsible lesions that cause ischemia in patients with cerebral ischemia. This is because collateral circulation via Willis ring and leptomeningeal anastomosis modifies blood circulation status. Even in individuals without cerebrovascular disorders, there is known to be large variation in perfusion territories of brain arteries.[9–11]

According to the guidelines, if symptomatic stenosis is greater than 70% in noninvasive images or 50% in angiography by North American Symptomatic Carotid Endarterectomy Trial (NASCET) method, it is an indication for vascular reconstruction.[12] Therefore, prior to the revascularization procedure, it is necessary to determine the responsible blood vessel of the ischemic lesion. However, according to the study by Hartkamp et al., 12 out of 149 (8%) infarcts were misclassified with standard assessments, using a perfusion atlas, and were not located in the original perfusion territory.[13] The gold standard of territory imaging is digital subtraction angiography(DSA), which provides detailed information about macrovascular blood flow and its territories. However, it is an invasive procedure with an increased risk for cerebral embolisms, especially in patients with steno-occlusive disease. As an alternative, the most effective method is selective labeling using MRI ASL, and in recent years, many such methods have been developed.

The early stage labeling technique required the placement of a thick slab in the plane to selectively label only the target artery.[3, 14]Improvements to this method haves been made through proof of consistency of the perfusion area and enhanced selectivity of labeling.[15, 16]

Superselective pCASL, currently the most sophisticated technique, uses a circular labeling spot and applies rotating gradients and phase changes in the pCASL labeling train radiofrequency pulses.^4^ The advantage of superselective pCASL is that it has more flexibility in placing labeling spots than slab plane labeling methods. Selective labeling is also possible in patients whose blood vessels are tortuous or prolonged.

The advantages and disadvantages of our method compared to the superselective pCASL method are as follows.

Image acquisition time per vessel is almost equal between the two methods at approximately 2 min, and image quality is also similar.

The absolute CBF value in the perfusion territories on the subtraction ASL images is inaccurate, as it depends on masking rate. Even in superselective pCASL, absolute CBF value is influenced by labeling efficiency, so it is similarly inaccurate. The territory area is correct in both methods, however, the absolute value of CBF should be carefully evaluated using the control ASL image in our method.

In our method, to increase the reproducibility of masking, it is necessary to comfirm the area of the artifact with the localizer image, which is not necessary in superselective pCASL. If the area of susceptibility artifact is too small to extend to the carotid artery or if it is large and affecting the vertebral artery, it is necessary to pull the subject out of the MRI bore to change the size and/or placement of the bolt and nuts. In addition to data collection, our method may take more time to employ, which is a disadvantage.

We should also consider the heat generation of the metal, in our method, which is not a factor in the superselective pCASL technique. Radiofrequency irradiation heats the metals to an extent that is linearly dependent on the specific absorption rate.[17] With the 3-tesla scanner, this rate is strictly controlled, so the risk of excessive heating is extremely low, but extreme caution is required to ensure that the metals do not touch the skin directly.

The advantage of hemilaterally masked ASL is that territory mapping images can be acquired with conventional ASL parameters and do not require advanced superselective labeling applications. This technique will be a great help for many MRI technicians and physicians who only have a conventional ASL application and would like to use territory mapping as a practical routine examination.

A limitation of this study includes has the comparison of our method and the superselective pCASL methods, which was not performed by a single MRI scanner. For strict comparison and confirmation of usefulness, further investigations need to be performed on the MRI scanner on which the superselective labeling application has been installed.

## Conclusions

Creating susceptibility artifacts with non-magnetic metals on the neck can mask spin labeling of the carotid artery and allow visualization of hemilateral ICA perfusion territories in hemilaterally masked ASL, just as we expected. This technique would be useful for evaluating perfusion areas in patients with carotid artery stenosis.

## Abbreviations

ASL: arterial spin labeling
CBF: cerebral blood flow
pCASL: pseudocontinuous arterial spin labeling
CeBF: cerebellar blood flow
